# Editorial: Current Analytical Trends in Drug Testing in Clinical and Forensic Toxicology

**DOI:** 10.3389/fchem.2021.673397

**Published:** 2021-05-07

**Authors:** Eugenia Gallardo, Mário Barroso, Marta Concheiro-Guisan, Ana de-Castro-Ríos

**Affiliations:** ^1^Health Sciences Research Center, Faculty of Health Sciences, University of Beira Interior, Covilhã, Portugal; ^2^Instituto Nacional de Medicina Legal e Ciências Forenses (INMLCF), Lisbon, Portugal; ^3^John Jay College of Criminal Justice, New York, NY, United States; ^4^Institute of Forensic Sciences “Luis Concheiro”, University of Santiago de Compostela, Santiago de Compostela, Spain

**Keywords:** drugs of abuse, chromatographic techniques, drug testing, clinical and forensic toxicology, trends in bioanalysis, novel analytical approaches, new psychoactive substance

The articles included in this collection cover novel analytical approaches, including chromatographic and spectrometric methods, and sample preparation techniques for the investigation and analysis of several classes of compounds. These compounds include novel psychoactive substances (NPS) as well as other drugs and substances within the scope of clinical and forensic toxicology, and other fields, such as doping control.

Current trends in bioanalysis require the constant development of novel analytical tools, which includes efficient sample collection procedures and adequate sample preparation protocols in order to maximize compound detection, even at trace levels. Taking into account that the number of substances possibly present in a sample are increasing, efficient multi-analyte methods are usually necessary. The detection of NPS, including synthetic cathinones and synthetic cannabinoids, is becoming more and more important as several reports of acute intoxications and deaths are often being issued. Therefore, developing new analytical methods and strategies help scientists efficiently face those challenges, allowing laboratories to be one-step ahead.

In this topic collection, four publications focus on the investigation of different critical aspects of NPS. These studies provide new tools for the identification of new NPS derivatives and metabolites (Frison et al.; Lopes et al.), investigate the stability of synthetic cathinones in biological samples and storage solvents (Ciallella et al.) or explore the detectability of synthetic cannabinoids in hair samples (Shi et al.).  Frison et al. described the analytical characterization, following two non-fatal intoxication cases, of 3-methylmethcathinone (3-MMC) and 3-methoxyphencyclidine (3-MeO-PCP) in seized products, and the investigation of 3-MeO-PCP and metabolites in biological samples. Three different analytical approaches were employed to identify 3-MMC and 3-MeO-PCP in seized materials, including gas chromatography-mass spectrometry (GC-MS) with electron impact ionization, liquid chromatography-high-resolution accurate-mass Orbitrap mass spectrometry (LC-HRAM-Orbitrap-MS), and solid deposition gas chromatography-Fourier transform infrared spectroscopy (sd-GC-FTIR). The role of the two latter techniques in attaining full structural characterization of the psychoactive drugs and related metabolites, in both non-biological and biological samples, was highlighted. The novelty of Frison et al. work lies in this aspect of the employment of LC-HRAM-Orbitrap-MS and sd-GC-FTIR instrumentation to identify and characterize new psychoactive substances in the absence of reference standards in different types of samples. Lopes et al. also employed high resolution mass spectrometry (HRMS) for the identification of new metabolites of synthetic cathinones. In particular, they identified the phase I and II metabolites of 4'-methyl-N,N-dimethylcathinone (4-MDMC), 4'-methyl-N,N-diethylcathinone (4-MDEC), 4'-chloro-α-pyrrolidinovalerophenone (4Cl-PVP) and 4'-chloroethylcathinone (4-CEC). The metabolites herein identified are expected to play an important role not only because they act as potential selective biomarkers of the intake of the studied synthetic cathinones, but also because their potential adverse effects may be better understood. In addition, those causative agents may be linked to toxicities, thereby helping understanding and treating non-fatal intoxications. This study highlights the critical role of high resolution mass spectrometry in the investigation of the toxicity of NPS.  Ciallella et al. studied the stability of four Schedule I synthetic cathinones, namely mephedrone, naphyrone, MDPV, and α-PVP. Indeed, stability is a critical parameter for toxicology laboratories. Understanding the variability in the analyte concentrations due to stability issues has an impact in the subsequent interpretation of concentration data derived from biological sample analysis. In this research, Ciallella et al. were able to analyze these cathinone derivatives employing solid phase extraction of blood and urine samples, and analyzing the compounds by GC-MS. The results of this study provided a comprehensive overview of the stability of these compounds in biological matrices over an extended period, including the evaluation of an alternative preservative and the inclusion of solvent-based working solutions. Shi et al. developed and validated a novel target analytical method for the determination of the synthetic cannabinoid 5F-MDMB-PICA and five metabolites in hair samples by liquid chromatography-tandem mass spectrometry (LC-MSMS). This new synthetic cannabinoid has been used in the form of “spice-like” herbal incenses or in electronic cigarette oil, and this study provides critical data for the interpretation of hair testing for this type of substances. The sensitivity of LC-MSMS allowed the authors of this study to achieve limits of detection at low pg/mg level in hair.

This topic collection also covers new challenges and strategies of analytical methods. Jurásek et al. investigated the potential of X-ray powder diffraction (XRPD) for rapid and simple identification of drugs of abuse in seized material. In this work, the authors proved that XRPD could be used to unambiguously identify 7 selected psychoactive substances (including 5 NPS) in different street sample mixtures, and proposed this technique as a complement to Infrared and Raman spectroscopies, the most common techniques used for this purpose, when unequivocal drug identification with these techniques is hindered by drug or additives native fluorescence or matrix complexity. Joye, Widmer et al. used matrix-assisted laser desorption/ionization (MALDI) high-resolution mass spectrometric (HRMS) technologies, which have been used to analyze the samples seized in the black market. The authors highlight the potential of MALDI-HRMS as high-throughput analytical strategy in toxicology laboratories, which significantly accelerates the detection and quantification of several drugs of abuse. The developed approach showed qualitative and quantitative results comparable to those obtained using LC-MS and GC-MS, reducing the analytical procedure by six times. With the development of bioinformatics tools and shared online libraries, new drugs of abuse that appear in the markets are easily identified and determined. In a second manuscript, Joye, Rocher et al. also used liquid chromatography hyphenated with Orbitrap high-resolution mass spectrometry with parallel reaction monitoring (PRM) for the quantification of the major classes of psychoactive substances present in the context of driving under the influence of drugs (DUID), such as cannabinoids, cocaine and its metabolites, amphetamines, opiates and opioids, and the major benzodiazepines and z-drugs, achieving the required sensitivity for DUID cases using a sample amount as low as 0.1 mL of whole blood. In addition to high resolution, Orbitrap-based PRM acquires all the selected precursor ions, avoiding *a priori* knowledge of the fragments of interest for method development, which represents an advantage over classical multiple reaction monitoring (MRM).  Habib et al. reviewed the strategies for chemical analysis of drugs of abuse and explosives, using mass spectrometry-based approaches. Several new ionization sources were revisited and the mechanisms of ion formation following their use were addressed for illicit drugs and explosives. The authors concluded presenting the main challenges that the future holds regarding the analysis of non-volatile compounds in what concerns ionization procedures.

Greener sample preparation techniques, like hollow fiber liquid-phase microextraction, are also presented in this collection, namely by de Oliveira Silveira et al., to determine the main markers of Ayahuasca consumption in urine specimens. This alternative and eco-friendly sample preparation approach was fully validated, showing excellent limits of detection and quantification (1-5 ng/mL), reproducibility, reduced matrix effect interferences, and outstanding recoveries (above 80%).

Finally, new approaches to determine drug use via wastewater analysis were reviewed by Zilles Hahn et al. The authors addressed new insights about wastewater-based epidemiology (WBE) as a useful tool to detect in real time illicit drug use by a population. Also, the most important biomarkers of drugs of abuse consumption in wastewater and the fundamentals of polar organic chemical integrative sampling (POCIS) in WBE were discussed and compared with other strategies.

In summary, this collection covers Research Topics representative of the recent trends and advances in drug testing and new compound identification in biological specimens, with focus on the development of novel analytical approaches, new chromatographic and spectrometric techniques, and sample preparation procedures, including miniaturized and environmentally friendly methodologies.

## Author Contributions

All authors listed have made a substantial, direct and intellectual contribution to the work, and approved it for publication.

## Conflict of Interest

The authors declare that the research was conducted in the absence of any commercial or financial relationships that could be construed as a potential conflict of interest.

